# Multiple areas investigation reveals the genes related to vascular bundles in rice

**DOI:** 10.1186/s12284-019-0278-x

**Published:** 2019-03-21

**Authors:** Cheng Fei, Xin Geng, Zhengjin Xu, Quan Xu

**Affiliations:** Rice Research Institute of Shenyang Agricultural University, Shenyang, 110866 China

**Keywords:** Rice, *indica/japonica* cross, Vascular bundles, *DEP1*, Abscisic acid

## Abstract

**Background:**

The vascular bundle in the panicle neck is a crucial trait in rice (*Oryza sativa*) production that differs between the *indica* and *japonica* subspecies. However, the effect of *indica/japonica* genetic background on the vascular bundles remains unknown.

**Results:**

A series of recombinant inbred lines (RILs) derived from a cross between *japonica* and *indica* were planted in three areas. High-throughput sequencing was conducted to determine the *indica* pedigree percentage and for quantitative trait locus (QTL) analysis. The *indica* pedigree affected the number of large vascular bundles (LVBs), but not the number of small vascular bundles (SVBs). QTL analysis identified a locus (*qLVB9*) that was pleiotropic for both LVBs and SVBs in all three areas, and *qLVB9* appeared synonymous with *DENSE AND ERECT PANICLE 1* (*DEP1*). Using CRISPR/Cas9 gene editing and gene overexpression technology, we confirmed that the truncated *dep1* allele increased the number of LVBs, and resulted in LVBs more closely associated to the *indica* pedigree. RNA sequencing showed that the truncated *dep1* allele downregulated the *AP2-like* gene family. The double mutant for the *DEP1* and *AP2-like* genes (*OsAP2–39*) showed decreased endogenous abscisic acid (ABA) level and insensitivity to exogenous ABA treatment, confirming that both *DEP1* and *OsAP2*–*39* are involved in the ABA response mechanism.

**Conclusions:**

The present study showed the *qLVB9/DEP1* affects LVBs, and involved in ABA signaling via regulating the *AP2-like* gene family. These results offer new insights into the function of *qLVB9/DEP1* in rice.

**Electronic supplementary material:**

The online version of this article (10.1186/s12284-019-0278-x) contains supplementary material, which is available to authorized users.

## Background

Rice is one of mankind’s major food staples. Due to the continuing growth of the global population and a decrease of availability of arable land, increasing grain yield is an important goal of scientists and rice breeders. In rice breeding, the selection of source, sink, and translocation capacity play critical roles in the improvement of rice yield potential (Donald, 1968, Lafitte and Travis, 1984, Ashraf et al., 1994). The vascular bundle, particularly that in the panicle neck, is the transport system that links the source to the sink and strongly affects the transport efficiency of photosynthetic products, mineral nutrients, and water (Peterson et al., 1982). Vascular bundles in plants play a significant role in the transportation of water, nutrients, and other substances required for biological processes (Lucas et al., 2013). There is a significant positive correlation between grain yield and the number of vascular bundles in rice (Ashraf et al., 1994), wheat (Evans et al., 1970), and oats (Peterson et al., 1982). The capacity of the vascular bundle system to efficiently transport various assimilates has been shown to be a limiting factor for improvements in rice production (Peterson et al., 1982).

The two subspecies of cultivated rice, namely *Oryza sativa ssp. indica* and *O. sativa* ssp. *japonica*, exhibit distinct differences in morphology and ecology. The *japonica* subspecies is mostly planted in higher latitudes, whereas *indica* is mainly distributed in lower latitudes (Garris et al., 2005). The vascular bundles are closely related to panicle traits, and there are significant differences of vascular bundles between rice subspecies (Fukuyama et al., 1999). The *indica* varieties have more vascular bundles in the neck panicles than that in *japonica*; moreover, the number of vascular bundles is significantly positively correlated with the number of rachis branches (Ling et al., 1982). The V/R (the ratio of the number of large vascular bundles to the number of primary branches) is an important parameter distinguishing *indica* from *japonica*, as the V/R is nearly one in *japonica* and approximately two in *indica* (Chen et al., 2007).

Quantitative trait locus (QTL) mapping has been utilized to elucidate the genetic mechanisms underlying various important agronomic traits. Over the past serveral decades, several QTLs associated with the responses of vascular bundles in the rice panicle neck have been identified (Sasahara et al., 2010, Zhang et al., 2002, Cui et al., 2003, Bai et al., 2012). Notably, *Accumulation of photosystem one 1* (*APO1*) (Terao et al., 2010) and *Narrow leaf 1* (*NAL1*) (Qi et al., 2008, Fujita et al., 2013) have been reported to participate in vascular bundle improvement.

Hybridization between *indica* and *japonica* rice for favorable trait selection has led to the development of high-yielding *japonica* rice in northern China. The introgression of the *indica* pedigree into the *japonica* genetic background significantly increased rice production (Sun et al., 2012). However, the effects of the *indica* and *japonica* genetic backgrounds on vascular bundle traits remain unclear. In the present study, the vascular bundle traits of a series of recombinant inbred lines (RILs) derived from a cross between *indica* and *japonica* that had been planted in three different areas were investigated. Combined with high-throughput resequencing, the *indica* pedigree percentage was determined for each RIL, and QTL analysis was conducted to detect the loci that were associated with vascular bundle responses.

## Results

### Characterization of the vascular bundles of the parent line and RILs from three different areas

**Fig. 1:**
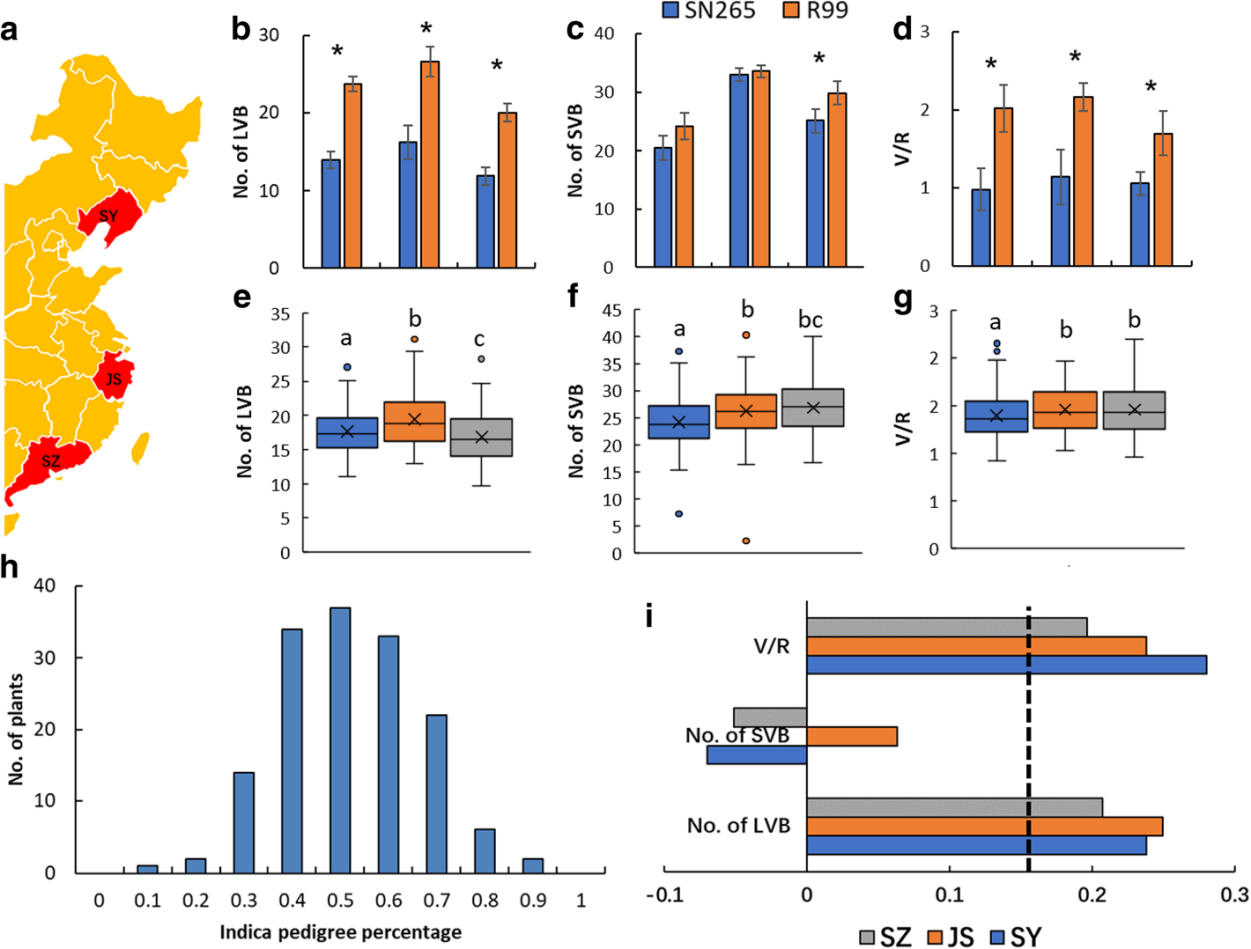
The vascular bundle characteristics of the parent lines and RILs in the three areas. **a** The locations of the three cultivated areas. **b**–**d** The vascular bundle characteristics of SN265 and R99 in the three areas. **e**–**g** The vascular bundle characteristics of the RILs in the three areas. * represents significance at the 5% level. **h** The distribution of the *indica* pedigree percentage of RILs. **i** The correlation efficiency of *indica* pedigree percentage to vascular bundles. The dotted lines indicate significance at the 5% level

To better understand the effect of the *indica*/*japonica* genetic background on vascular bundles in rice, we planted a series of 155 RILs derived from a cross between the *indica* variety “R99” and *japonica* variety “SN265” in three typical rice cultivation areas, namely Shenyang (SY) (N42°), Jiangsu (JS) (N32°), and Shenzhen (SZ) (N22°). The locations of the cultivation areas are shown in Fig. 1a. Then, we investigated the LVB, SVB, and V/R of the parent lines and RILs in each area. In the parent lines, a significant difference in the LVBs and V/R was observed between SN265 and R99 in all three areas, whereas a significant difference in SVBs between the parent lines was only detected in SZ (Fig. 1b-d). The RILs showed significant differences in LVBs among the three areas. The RILs in JS and SZ showed similar SVBs and V/R distributions, both significantly higher than that in SY (Fig. 1e-g). Analysis of variance (ANOVA) revealed differences among years, areas, and lines. In general, the variation among lines had the largest effect on LVBs, SVBs, and V/R, and significant differences were also observed among the areas, although no significant differences in vascular bundle traits were detected between 2016 and 2017 (Additional file 1: Table S1).

### The relationship between vascular bundles and yield components

To demonstrate the relationship between vascular bundles and yield components, we conducted a yield component analysis immediately after all of the lines in all three of the areas had reached maturity. The results showed that LVB, SVB, and V/R significantly affected grain number (Table 1). Moreover, LVB also affected the setting rate, whereas SVB was significantly negatively correlated with panicle number. Both the LVB and V/R showed a significant positive correlation with yield (Table 1). V/R regulated grain number in both the primary and secondary branches. Interestingly, LVB and SVB only affected grain number on the secondary branches but not on the primary branches, while LVB also only affected the grain-setting rate on the secondary branches (Table 1).

**Table 1:** The correlation efficiency of vascular bundles to yield components

Areas	Traits	NP	NG	NGPB	NGSB	ST	STPB	STSB	Yield
SY	LVB	−0.143	0.489^a^	0.101	0.344^a^	−0.185^a^	−0.050	− 0.206^a^	0.183^a^
	SVB	−0.305^a^	0.370^a^	0.108	0.298^a^	−0.076	−0.077	− 0.120	0.101
	V/R	0.025	0.325^a^	0.238^a^	0.390^a^	−0.123	−0.054	− 0.150	0.218^a^
JS	LVB	−0.074	0.519^a^	0.127	0.4048	−0.333^a^	−0.127	− 0.263^a^	0.208^a^
	SVB	−0.192^a^	0.358^a^	0.110	0.326^a^	−0.088	0.048	−0.117	0.105
	V/R	−0.019	0.335^a^	0.238^a^	0.393^a^	−0.119	−0.136	− 0.139	0.204^a^
SZ	LVB	−0.138	0.460^a^	0.019	0.238^a^	−0.223^a^	−0.076	− 0.265^a^	0.233^a^
	SVB	−0.370^a^	0.457^a^	0.073	0.242^a^	−0.124	−0.044	− 0.143	0.093
	V/R	0.069	0.262^a^	0.201^a^	0.159^a^	−0.107	−0.010	− 0.088	0.208^a^

*NP* number of panicle, *NG* number of grain per panicle, *NGPB* number of grain on primary branch, *NGSB* number of grain on secondary branch, *ST* setting rate, *STPB* setting rate on primary branch, *STSB* setting rate on secondary branch, ^a^, significant at 0.05 level

### The effects of the *indica*/*japonica* genetic background on vascular bundles

To elucidate the effects of the *indica*/*japonica* genetic background on vascular bundles, we conducted high-throughput sequencing of the RILs. The genomes of 155 RIL lines were sequenced to an average approximate depth of 6.25-fold on an Illumina HiSeq 2500 instrument, and the depth of the parent lines reached 30-fold. The high-throughput sequencing detected a total of 1,794,441 SNPs between SN265 and R99. Based on the study of divergence of 517 rice landraces in Huang et al. (Huang et al., 2010), totally 100,529 subspecies-specific SNPs that were the same in all of the *japonica* samples, but were different in *indica* were selected. We matched the 100,529 subspecies-specific SNPs to the 1,794,441 SNPs between SN265 and R99, and a total of 61,920 SNPs were merged. The 61,920 SNPs were then used for *indica* pedigree percentage analysis. In the present study, we defined the *indica* pedigree percentage as the ratio of the number of *indica* type SNPs to all 61,920 of the subspecies-specific SNPs, and the results showed that the *indica* pedigree percentage of the RILs followed a normal distribution (Fig. 1h). We further investigated the relationship between *indica* pedigree percentage and vascular bundles, the results showed that the *indica* pedigree percentage was positively correlated to V/R and the number of LVBs in all three of the areas, whereas the effect of the *indica* pedigree percentage on SVB differed among the three areas, and the correlation was not significant (Fig. 1i). Thus, the *indica* pedigree percentage mainly affects LVBs.

### QTL analysis of the vascular bundles among the three areas

**Fig. 2:**
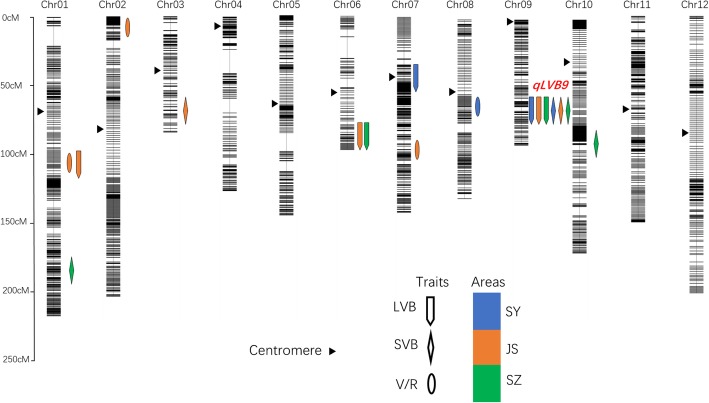
QTL analysis of vascular bundle traits based on high-density bin maps derived from the RIL population. The scale for the genetic length of each chromosome is shown on the left. The different colors indicate the QTLs detected in the different areas

**Fig. 3:**
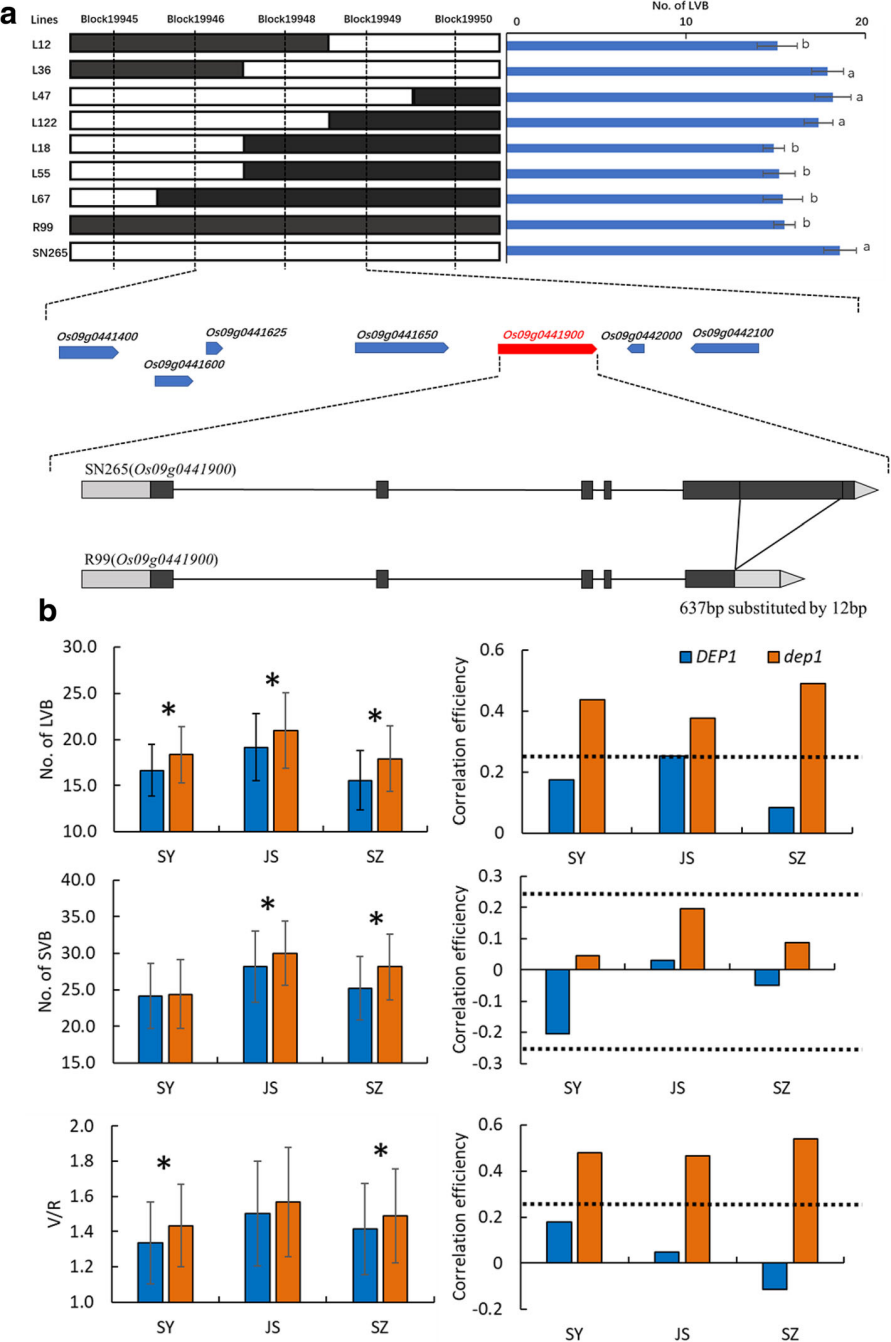
The fine mapping of *qLVB9* and the function change of *qLVB9.* **a** The candidate gene *qLVB9* was identified as *Os09g0441900/DEP1*. **b** The different functions of the normal *DEP1* allele and truncated *dep1* allele among three areas, and the correlation efficiency between vascular bundles traits and *indica* pedigree of these two alleles in three areas. * represents significance at the 5% level, the dotted lines indicate significance at the 5% level

We used the 1,794,441 SNPs between SN265 and R99 for QTL analysis. We namedd the SNPs that co-segregated with one another as “bins,” and a total of 3333 bins were used to construct the molecular linkage map using Highmap software. The phenotypic data of the LVBs, SVBs, and V/R of the three areas were used for the QTL analysis. A total of 17 QTLs for all of the traits were mapped independently on rice chromosomes 1, 2, 3, 6, 7, 8, 9, and 10 (Fig. 2 and Additional file 2: Table S4). Notably, one QTL cluster, *qLVB9*, was highlighted*.* The *qLVB9* cluster was pleiotropic for both LVBs and SVBs and was detected in all three of the areas. To further identify the exact gene responsible for *qLVB9*, the number of LVBs in SZ was selected and used in fine-mapping. We mapped the candidate gene to a 43-kb interval in block 19,948 (Fig. 3a). There were seven annotated genes in this bin. One of these genes, *DENSE AND ERECT PANICLE 1* (*DEP1*), has been previously shown to be a regulator for erect panicle architecture, grain number and nitrogen utilization. We subsequently conducted sequence analysis, and found that SN265 had a replacement of a 637-bp stretch in the exon 5 by a 12-bp sequence (Fig. 3a). To verify the effect of *DEP1* on vascular bundles, the RILs were further divided into two groups, the *DEP1* group (R99 type) and the *dep1* group (SN265 type). We found that the number of LVBs and SVBs in the *dep1* group was significantly higher than that in the *DEP1* group in all three areas. The V/R of the *dep1* group was also higher than that of the *DEP1* group, but only that in SZ was significant (Fig. 3b and Additional file 3: Table S2). In addition, the correlation between the *indica* pedigree percentage and the vascular bundle traits in the *dep1* group was markedly higher than that in the *DEP1* group (Additional file 3: Table S2). Taken together, these findings indicate that *DEP1* may be a major pleiotropic locus for both LVBs and SVBs, and furthermore, that the vascular bundles appear to be closely related to the *indica* pedigree percentage under the *dep1* genetic background than the *DEP1* genetic background.

### Verification by CRISPR/Cas9 gene editing technology and gene overexpression

**Fig. 4:**
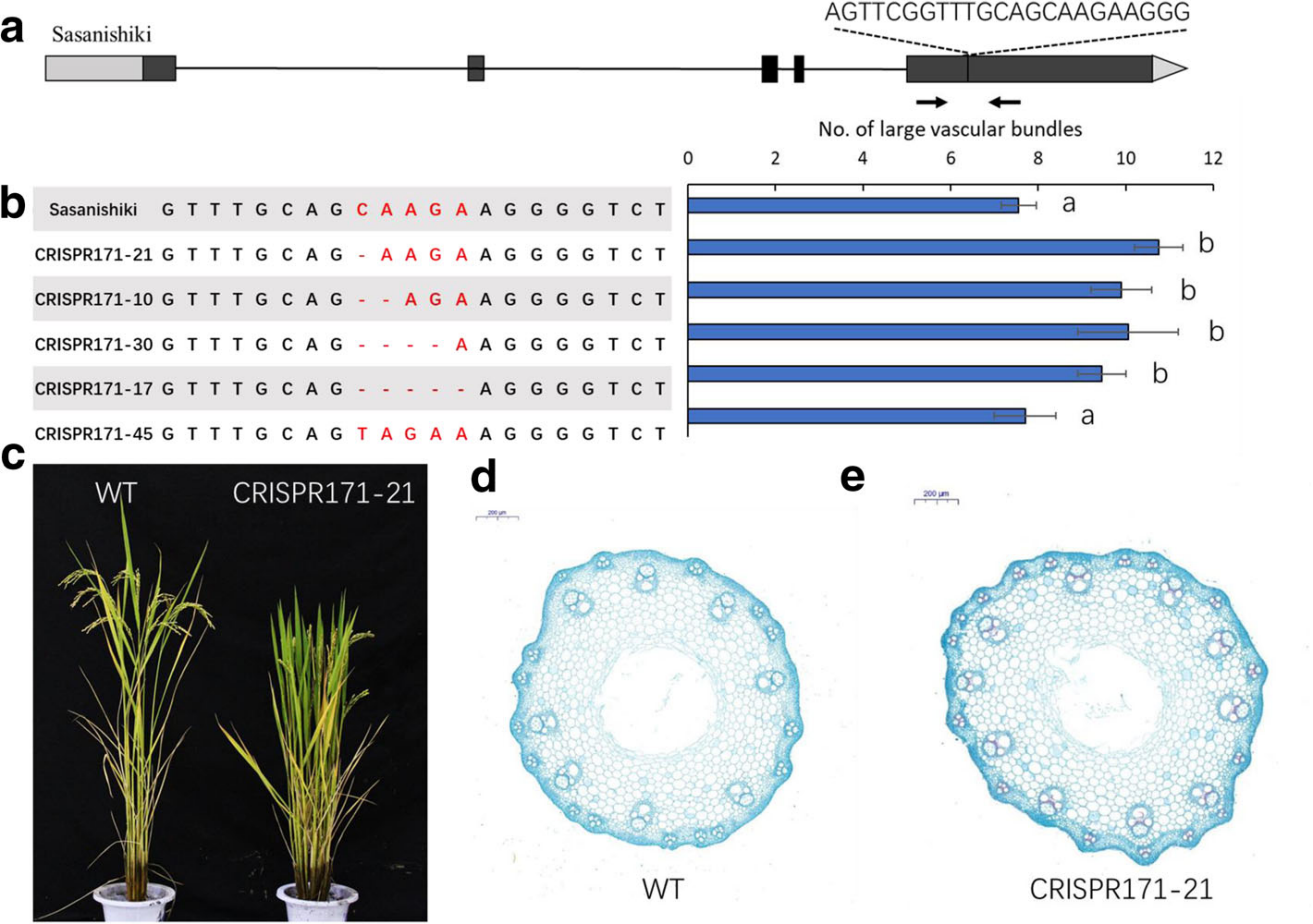
The CRISPR/Cas9-induced *DEP1* mutant lines and LVB investigation. **a** Schematic map of the genomic region of *DEP1* and the sgRNA target site. Arrows show the position of the PCR primers used for mutation detection. **b** The sequence of the mutant lines and the number of LVBs of the mutant lines. **c** The WT plant and CRISPR/Cas9 induced *DEP1* mutant line. **d** The difference in vascular bundles between WT plants and the CRISPR/Cas9 induced *DEP1* mutant line

We used CRISPR/Cas9 technology to confirm that *DEP1* is a candidate gene for *qLVB9* in the *japonica* cultivar Sasanishiki. The sgRNA was designed in the middle of exon 5, similar to SN265 (Fig. 4a). At least 30 independent regenerated transgenic lines were obtained. We sequenced the T_2_ mutants, and found five homozygous mutants (Fig. 4b). The deletion in exon 5 of four mutant lines was predicted to result in a frame shift. The sequence substitution in one mutant line was predicted to lead to an amino acid change. The number of LVBs in the four frame shift mutations was significantly increased compared to Sasanishiki, whereas the substitution line showed a similar number of LVBs to that in Sasanishiki (Fig. 4b). The details concerning agronomic traits of WT and CRISPR/Cas9 gene edited plants ware given in Additional file 4: Table S5. We further constructed a *DEP1* overexpression plant to verify the function of *DEP1* in vascular bundle regulation. The full-length cDNA (SN265) with a 35S promoter was introduced into SN265 (Additional file 5: Figure S1). Among the transgenic lines, TL35 and TL44 exhibited a significant increase in *DEP1* expression compared to the wild-type. The number of LVBs in these two lines was also significantly increased (Additional file 5: Figure S1). Thus, we concluded that *DEP1* regulates vascular bundle traits.

### RNA-Seq identifies *DEP1*-regulated genes

To establish the regulatory function of *DEP1*, RNA-Seq analysis was performed using total RNA extracted from wild-type (Sasanishiki) and CRISPR/Cas9-gene edited plants CRISPR171–21(*dep1-crispr*) at the heading stage, as CRISPR171–21 had the highest number of LVBs among the mutant lines. High-throughput sequencing using the Illumina HiSeq 2500 platform generated approximately 48.345 Gb of clean data. Furthermore, a total of 76 differentially expressed genes (DEGs) were detected between the wild-type and CRISPR171–21 plants, including 50 that were downregulated and 26 that were upregulated in CRISPR171–21. The Gene Ontology (GO) analysis and hierarchical clustering results of the DEGs are presented in Additional file 6: Table S6 and Additional file 7: Figure S2. Four *AP2* (*APETALA-2-Like transcription factor gene*) genes were enriched in DEGs. We conducted a gene expression analysis for the *AP2* genes, and the results showed that *Os04g0610400* exhibited the largest difference between CRISPR171–21 and the wild-type among the *AP2* genes. *Os04g0610400*/*OsAP2–39* has previously been shown to control abscisic acid (ABA) levels in rice (Yaish et al., 2010). In addition, we subsequently conducted expression profiling of various organs; both *DEP1* and *OsAP2–39* exhibited the highest expression levels in the rachis meristems (Additional file 8: Figure S3). Thus, we selected *OsAP2–39* for the subsequent experiments.

### *DEP1* involved ABA signaling via *OsAP2–39*

**Fig. 5:**
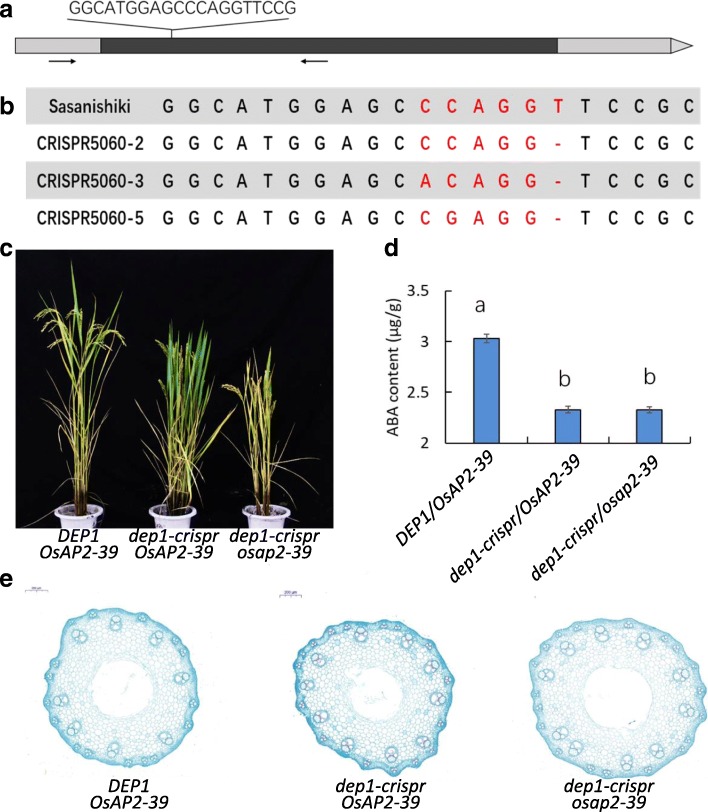
The endogenous ABA level and vascular bundles of WT and CRISPR/Cas9-induced *DEP1* mutant lines. **a** Schematic map of the genomic region of *OsPA2–39* and the sgRNA target site and the sequences of the mutant lines. The arrows show the position of the PCR primers used for mutation detection. **b** The sequences of the mutant lines. **c** The WT plant and CRISPR/Cas9 induced *DEP1* mutant line. **d** The endogenous ABA levels of WT and mutant lines. **e** The difference in vascular bundles between WT plants and the CRISPR/Cas9 induced *DEP1* mutant line

To confirm the relationship between *DEP1* and *OsAP2–39*, we generated a double mutant for *DEP1* and *OsAP2–39* using CRISPR/Cas9 technology under the genetic background of CRISPR171–21 (carrying *dep1-crispr* allele). We obtained three independent mutant lines (CRISPR5060–2, CRISPR5060–3, and CRISPR5060–5), the sequences for which are shown in Fig. 5b. We compared the endogenous ABA levels of the WT, *dep1-crspr* mutant and *dep1-crspr /osap2–39* double mutant. The endogenous ABA level of WT was significant higher than those of *dep1-crspr* mutant and *dep1-crspr /osap2–39* double mutant (Fig. 5d). We then compared the ABA response of *DEP1* and *OsAP2–39*. The seeds of three genotypes, namely the wild-type (*DEP1/OsAP2–39*), *dep1* single mutant CRISPR171–21 (*dep1-crspr /OsAP2–39*), and double mutant CRISPR5060–2 (*dep1-crspr /osap2–39*), were sown on sterile filter paper in Petri dishes supplemented with different concentrations of ABA. A significant delay in germination was observed for all of the seeds compared with the control. However, the germination of *DEP1/OsAP2–39* was more sensitive to exogenous ABA treatment than *dep1-crspr /OsAP2–39* and *dep1-crspr /osap2–39*. The double mutant *dep1-crspr/osap2–39* demonstrated the best germination in the ABA treatment (Additional file 9:Figure S4). In combination, these findings indicate that the truncated *dep1* participates in ABA signaling by downregulating *AP2-like* genes. We then investigated whether ABA was involved in the formation of LVBs. We applied exogenous ABA (10 μM) into irrigation water at the panicle initiation stage (about 35 days before heading) (Yuan et al., 2009). Then we recorded the number of LVBs 3 weeks after ABA application. The results showed that the plants with exogenous ABA applied slightly decreased the number of LVBs compared with the control plants. And the exogenous ABA decreased the expression level of *OsAP2–39* compared with control plants (Additional file 10: Figure S5).

## Discussion

*Indica* and *japonica* are two subspecies of Asian cultivated rice that can be differentiated in terms of vascular bundle architecture. *Indica* varieties tend to have more LVBs and a higher V/R ratio. The V/R of *indica* ranges from 1.6 to 2.0, whereas that of *japonica* is around 1.0. The increase in V/R ratio in *japonica* may be a mechanism for improving yield (Fukuyama et al., 1999). Our study confirmed that LVB numbersand V/R in *indica*-type lines are significantly higher than in *japonica*-type lines, whereas no distinct differences in SVBs were observed between the two subspecies. In *japonica*-type cultivars, each of the LVBs is directly connected to a primary branch. In *indica*-type cultivars, some LVBs are directly connected to the secondary rachis branches because there is more than one LVB in one primary branch (Fukushima and Akita, 1997, Terao et al., 2010). Our study showed that LVBs largely affect the grain number and setting rate of the secondary branches, but not that of the primary branches.

In this study, we detected a pleiotropic QTL (*qLVB9*) for both LVBs and SVBs in all three areas, and subsequent positional cloning and genetic complementation revealed that *qLVB9* is synonymous with *DEP1*; a gene that has been previously shown to regulate rice panicle architecture and grain number (Huang et al., 2009, Wang et al., 2009). *DEP1* has a modular arrangement with a conventional plant-specific Gγ subunit protein domain at its N-terminus, followed by a cysteine-rich domain at the C-terminus (Huang et al., 2009, Sun et al., 2014). G protein signaling participates in various growth and developmental processes in plants and animals (Temple and Jones, 2007, Xu et al., 2016). Thus, variations in G proteins may severely affect different important agronomic traits. However, RNA-Seq analysis has only identified a few DEGs (76 genes) in response to changes in *DEP1*. Nevertheless, we found *AP2-like* genes that were downregulated in CRISPR171–21. The overexpression of *OsAP2–39* leads to a reduction in yield by decreasing grain number per panicle (Yaish et al., 2010). Thus, the truncated *dep1* may increase grain number per panicle by downregulating the expression of *OsAP2–39*. Moreover, the overexpression of *OsAP2–39* caused an increase in the expression of a key ABA biosynthetic gene, *OsNCED-1*, which subsequently led to an increase in endogenous ABA levels. ABA signaling influences vascular and bundle sheath cells in *Arabidopsis* (Galvezvaldivieso et al., 2009). Recent studies have demonstrated that *OsAP2–39* is also involved in drought tolerance (Wan et al., 2011), and *qPE9–1* (also synonymous with *DEP1*) and *RGB1* play distinct roles in ABA responses and drought adaptation (Zhang et al., 2015). Thus, both *DEP1* and *OsAP2–39* have been shown to be involved in ABA signaling and drought responses. In this study, the ABA treatment for *DEP1/OsAP2–39*, *dep1-crispr/OsAP2–39,* and *dep1-crispr/osap2–39* plants also demonstrated that both *DEP1* and *OsAP2–39* are involved in the ABA response mechanism, and these two genes may work in the same pathway.

Previous studies have shown that the *dep1* allele results in an increase in grain number and a decrease in setting rate by regulating the *Gn1a* (Huang et al., 2009, Ashikari et al., 2005). Our study has revealed that the truncated *dep1* can significantly increase the number of both LVBs and SVBs. LVBs were significantly positively correlated with grain number and significantly negatively correlated with setting rate (Table 1). These results may explain the phenotype associated with an increase in grain number and a decrease in setting rate in *dep1* plants. We determined that plants with a truncated *dep1* are significantly more sensitive to *indica* pedigree percentage than the wild-type *DEP1* plants. In *dep1* plants, the number of LVBs significantly increased with the increase in *indica* pedigree percentage, whereas no significant change was observed in the *DEP1* plants. Moreover, the *indica* pedigree percentage was negatively correlated with SVBs in *DEP1* plants, but significantly positively correlated with SVBs in *dep1* plants. The new function of *DEP1* may present a strategy for breeding under the *indica* genetic background.

In addition to *qLVB9/DEP1*, our study also detected a QTL (*qLVB6*) for LVB within the 28.45–28.83 Mb interval on chromosome 6 in JS and SZ. In this region, *Os06g0685700*, which is similar to the auxin response factor gene, is considered to be the candidate gene of *qLVB6*. Transgenic plants of *Os06g0685700* exhibit pleiotropic defects in growth and development (Huang et al., 2016), and a genome-wide association study also predicted that *Os06g0685700* is a LVB-regulating gene (Zhai et al., 2018). Sequence analysis detected several SNPs and InDels at exon regions between SN265 and R99 (Additional file 11: Figure S6), but the function of *Os06g0685700* in the vascular bundles requires further investigation. The previous studies demonstrated that *APO1* (Terao et al., 2010) and *NAL1*(Qi et al., 2008, Fujita et al., 2013) are participate in vascular bundle improvement. Our study detected the QTLs corresponding to LVBs at Chr. 6 in JS and SZ, which was consistent with the *APO1*. However, the *NAL1* locus was absent in the QTL analysis of LVB. Then we compare the sequence of *NAL1* between SN265 and R99. The results showed that the sequence of *NAL1* in SN265 was identical to Nipponbare, and the sequence of *NAL1* in R99 was identical to 93–11. We believe that the *NAL1* might mainly effect the number and the distribution pattern of LVBs in leaves, or the effect of *NAL1* may be weaker under some particular genetic background. Nevertheless, more experiments concerning relationship among *NAL1, APO1, DEP1,* and *OsAP2–39* would be interest.

## Conclusions

Vascular bundle is an important trait in rice production, and a highly significant difference was found between the two subspecies, *indica* and *japonica*. However, the effect of an *indica/japonica* genetic background ono the vascular bundles remained unknown. The present study showed that *qLVB9/DEP1* has a large effect on the number of LVBs, and the vascular bundles of plants harboring the truncated *qLVB9/DEP1* allele were more sensitive to the alteration of ecological conditions and genetic background compared to plants that carried the normal *qLVB9/DEP1* allele. Moreover, *qLVB9/DEP1* was involved in ABA signaling via regulating the *AP2-like* gene family. These results offer new insights into the function of *qLVB9/DEP1* in rice.

## Methods

### Plant materials

A total of 155 RILs derived from a cross between ‘Shennong265’ (*O. sativa* L. ssp. *japonica*) and ‘R99’ (*O. sativa* L. ssp. *indica*) were used in this study. This RIL population was developed from a single-seed descendant that had been inbred for over 10 generations. Field experiments were conducted in three typical rice cultivation areas: the Rice Research Institute of Shenyang Agricultural University (SY) (N41°, E123°), the sub-base of the China National Hybrid Rice R&D Center in Jiangsu Province (JS N32°, E120°), and the Agricultural Genomics Institute at Shenzhen (SZ) (N22°, E114°) for two growing seasons in 2016–2017. Cultivation methods and field management were described in our previous report (Li et al., 2018b) (Additional file 12: Table S3). The paddies were harvested 45 days after heading for each line in each of the three areas.

### Evaluation of vascular bundles, yield components and ABA level

We evaluated the vascular bundles after the flowering stage. The main stems with spikes were sampled from eight plants per RIL. The method of vascular bundle evaluation was conducted as described in Liu et al. (2016). At the mature stage (35 days after flowering), the aboveground portions of eight plants per RIL were harvested from each plot. The yield components measurement was conducted as described in Li et al. 2018a (Li et al., 2018b). The evaluation of endogenous level and exogenous ABA response was conducted as described in Yaish et al. (2010).

### Genome resequencing and QTL mapping

The genomic DNA of the RILs together with their parents (SN265 and R99) was isolated using the cetyltrimethyl ammonium bromide (CTAB) method (Murray and Thompson, 1980). Sequencing libraries were constructed from these samples and sequenced on the Illumina HiSequation 2500 system according to the manufacturer’s instructions. The detail are described in our previous study (Li et al., 2018a).

### Vector construction and plant transformation

We conducted the CRISPR/Cas9 gene editing vector construction as described by (Li et al., 2017). The 23-bp targeting sequences (including PAM) were selected within the target genes, and their targeting specificity was confirmed using a BLAST search against the rice genome (Hsu et al., 2013) Rice transformation was performed as described elsewhere (Nishimura et al., 2006). Genomic DNA was extracted from these transformants, and primer pairs flanking the designed target site were used for PCR amplification. The PCR products (300–500 bp) were sequenced directly and identified using the Degenerate Sequence Decoding method (Ma et al., 2015). The cDNA was cloned into the pBWA(V) HS vector between the 35S promoter and terminator, generating a *35S::DEP1* construct (Additional file 5: Figure S1).

### RNA sequencing (RNA-Seq) analysis

Total RNA (10 mg) was extracted from young panicles of the CRISPR171–21 and wild-type plants at the heading stage, and three CRISPR171–21 or wild-type biological samples were pooled together. We conducted the RNA-Seq library preparation and DEGs analysis according to the method described in (Li et al., 2015).

## Additional files

Additional file 1: **Table S1.** ANOVA analysis of year, area, and lines for LVBs, SVBs, and V/R (PPTX 65 kb)

Additional file 2: **Table S4.** The QTLs information of three areas (PPTX 78 kb)

Additional file 3: **Table S2.** The effect of the *DEP1/dep1* allele on LVBs, SVBs, and V/R (PPTX 58 kb)

Additional file 4: **Table S5**. The agronomic traits of WT and gene edited plants (PPTX 80 kb)

Additional file 5: **Figure S1.** The overexpression of *DEP1.* (a) Overexpression construct for rice transformation. (b) The expression levels of TL35, TL44, and wild-type (WT). (c) The number of LVBs of TL35, TL44, and WT. (d) The endogenous ABA level of WT and *dep1* overexpression lines. (e) The WT plant and the *dep1* overexpression lines. (f) The difference in vascular bundles in WT plant and *dep1* overexpression lines. (PPTX 53 kb)

Additional file 6: **Table S6.** The RNA-seq information (PPTX 146 kb)

Additional file 7: **Figure S2.** Expression patterns of DEGs. (a) Hierarchical clustering of DEGs. (b) GO analysis of the DEGs between CRISPR171–21 and wild-type plants. (c) The expression level of *AP2-like* genes in CRISPR171–21 and wild-type plants. (PPTX 3444 kb)

Additional file 8: **Figure S3.** *DEP1* and *OsAP2–39* expression levels in various organs of Sasanishiki. (PPTX 561 kb)

Additional file 9: **Figure S4.** Germination investigation under exogenous ABA treatment. (PPTX 52 kb)

Additional file 10: **Figure S5.** The number of LVBs between control and ABA treatment plants, and the expression level of *OsAP2–39* between control and ABA treatment plants. (PPTX 945 kb)

Additional file 11: **Figure S6.** *Os06g0685700* sequence differences between SN265 and R99 based on the sequence of SN265. (PPTX 46 kb)

Additional file 12: **Table S3.** The fertility of the soil in three areas (PPTX 57 kb)
