# Supplementary figures and images for: Multiple areas investigation reveals the genes related to vascular bundles in rice
Source: Rice (N Y). 2019 Mar 21;12:17. doi: 10.1186/s12284-019-0278-x (PMC6428884; doi:10.1186/s12284-019-0278-x)

## Slide 1
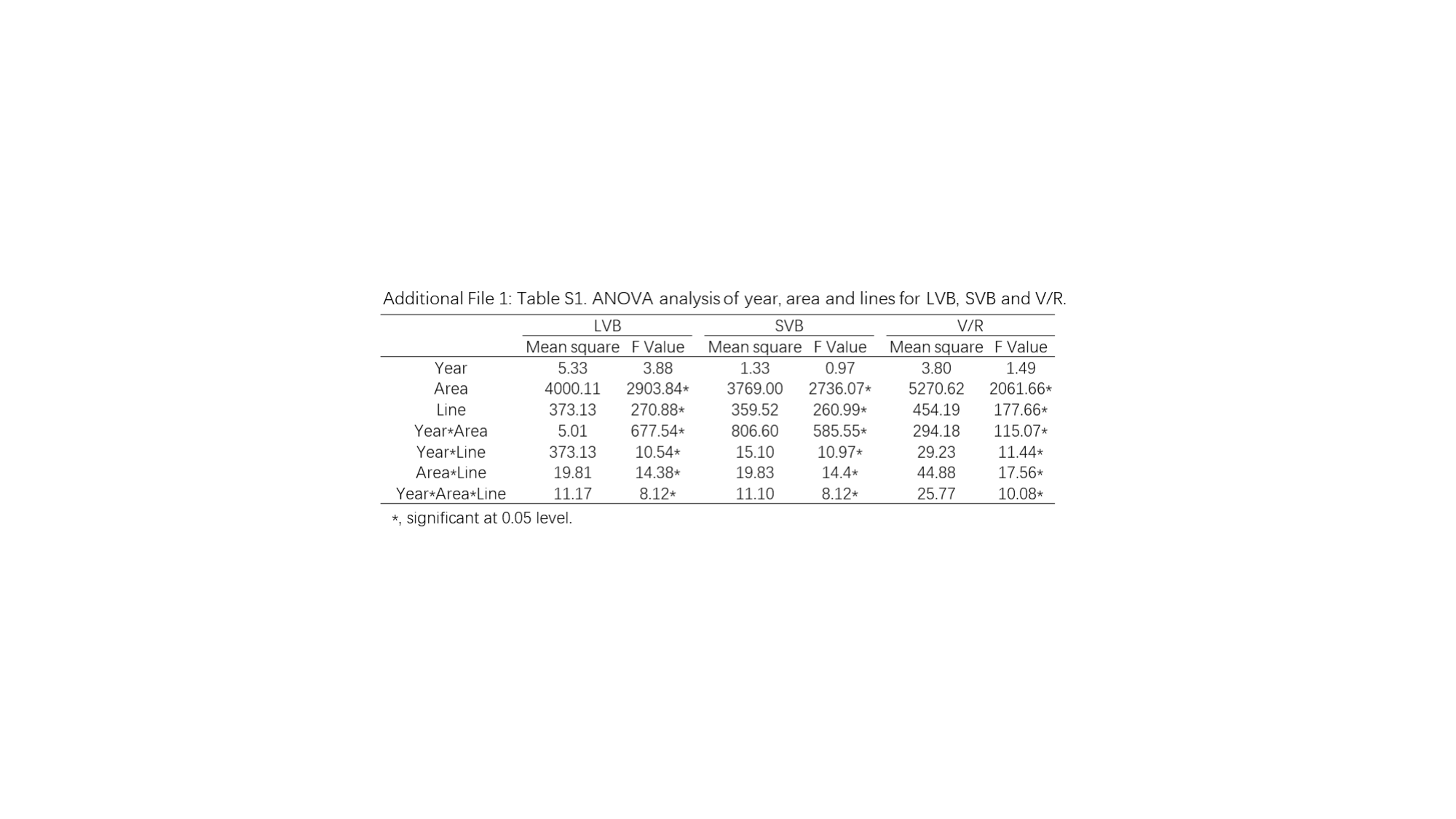

Supplement: Supplementary file 1 — Table S1. ANOVA analysis of year, area, and lines for LVBs, SVBs, and V/R (PPTX 65 kb) [file 12284_2019_278_MOESM1_ESM.pptx]

## Slide 1
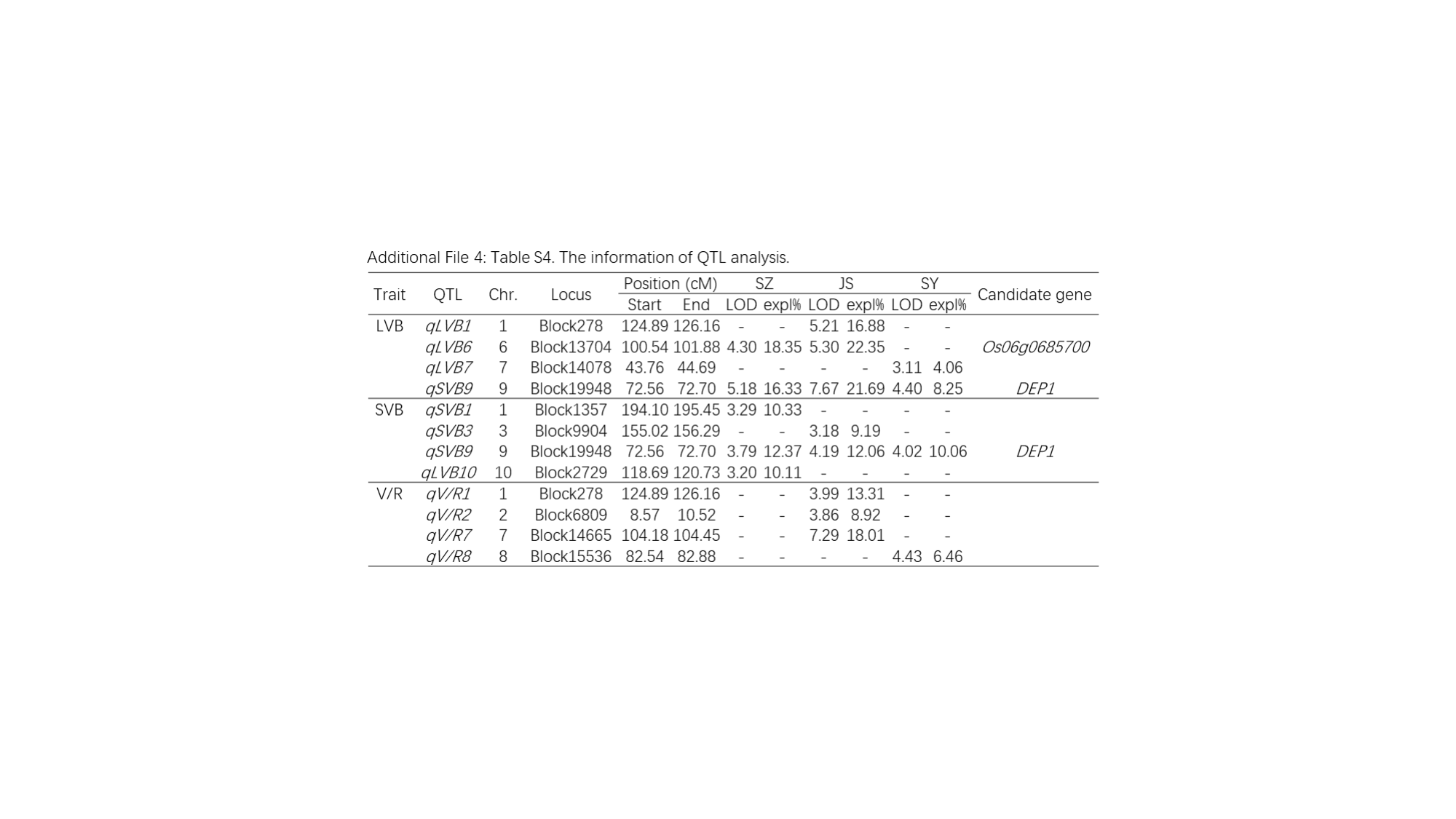

Supplement: Supplementary file 2 — Table S4. The QTLs information of three areas (PPTX 78 kb) [file 12284_2019_278_MOESM2_ESM.pptx]

## Slide 1
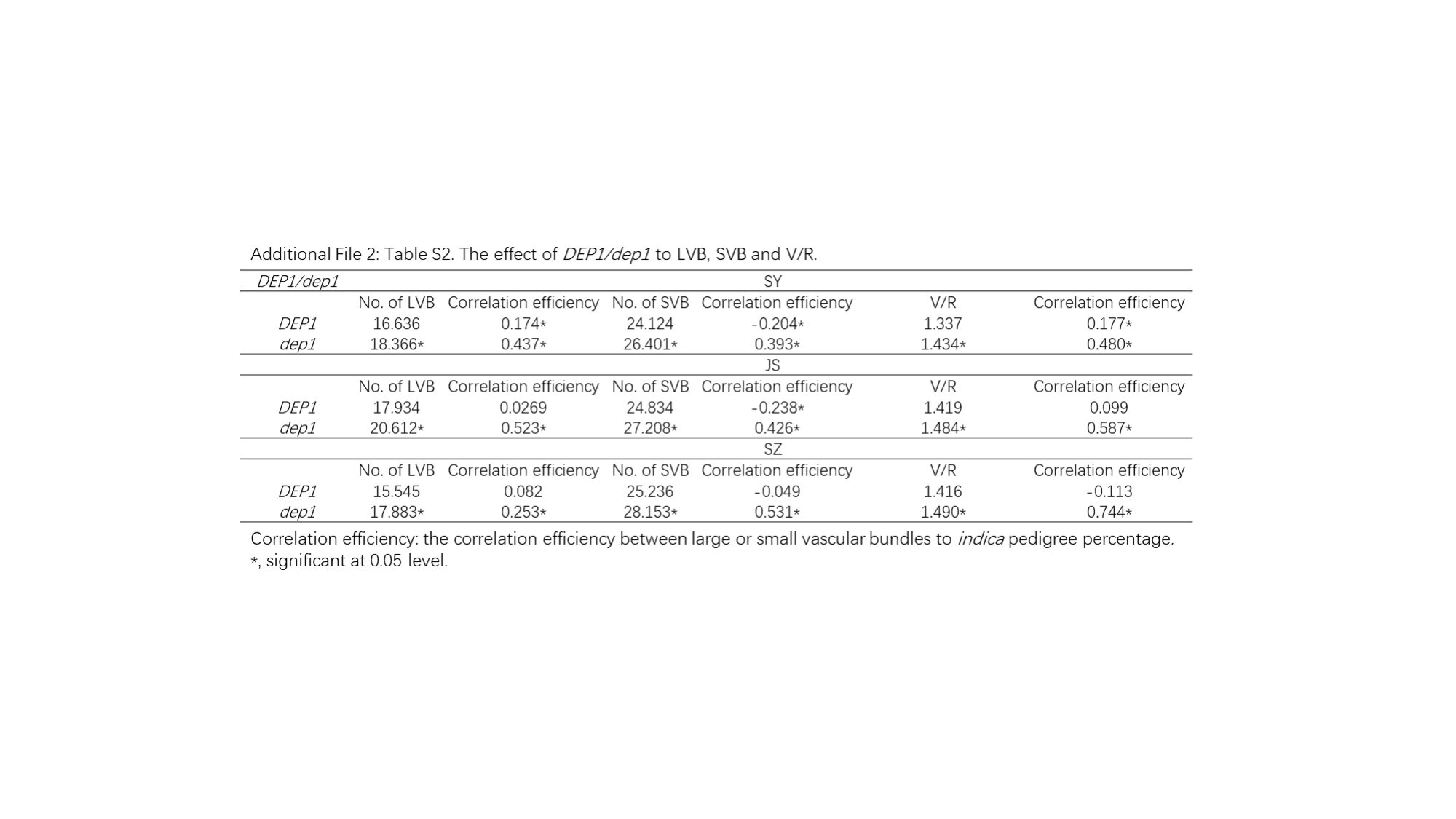

Supplement: Supplementary file 3 — Table S2. The effect of the DEP1/dep1 allele on LVBs, SVBs, and V/R (PPTX 58 kb) [file 12284_2019_278_MOESM3_ESM.pptx]

## Slide 1
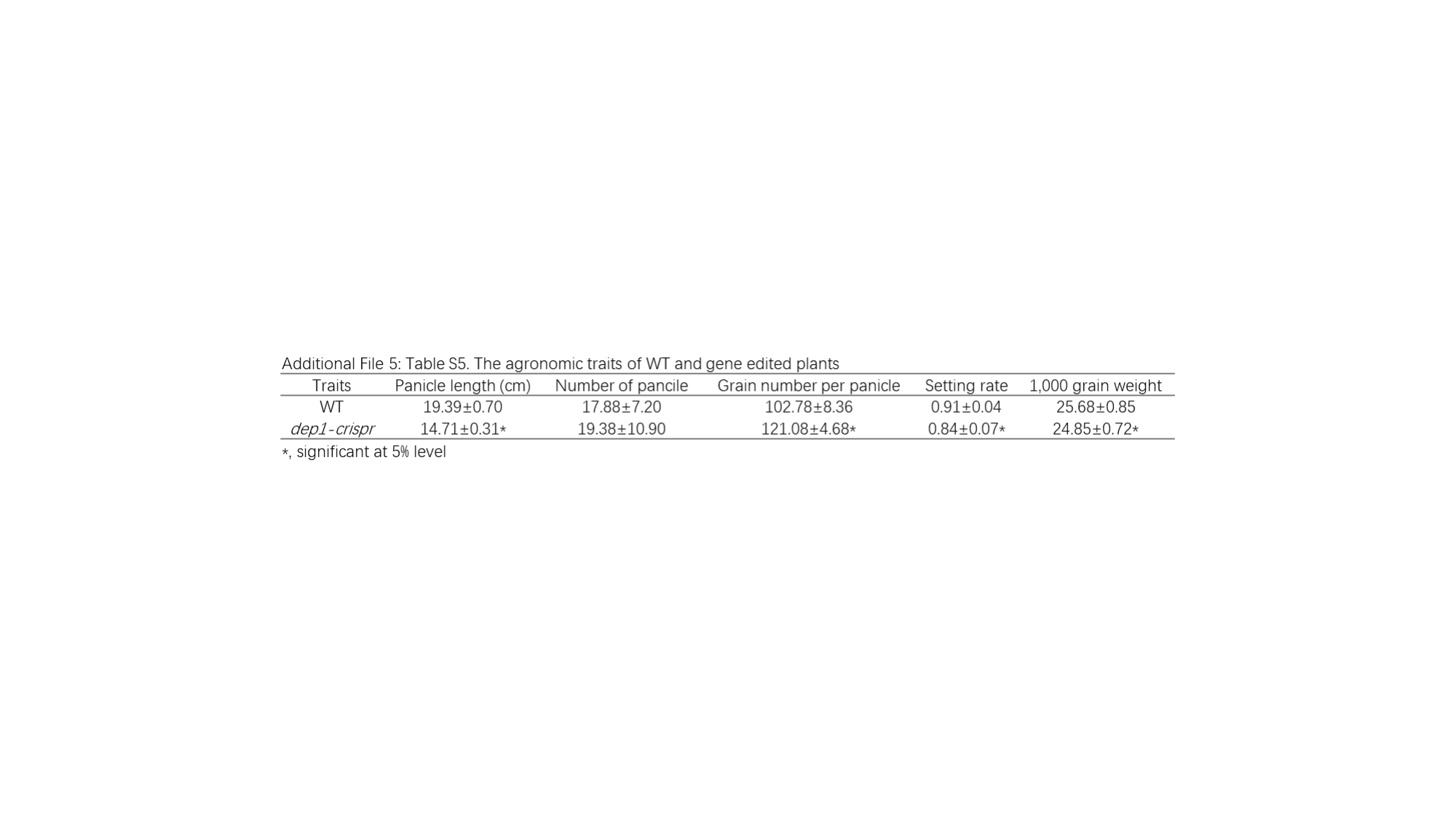

Supplement: Supplementary file 4 — Table S5. The agronomic traits of WT and gene edited plants (PPTX 80 kb) [file 12284_2019_278_MOESM4_ESM.pptx]

## Slide 1
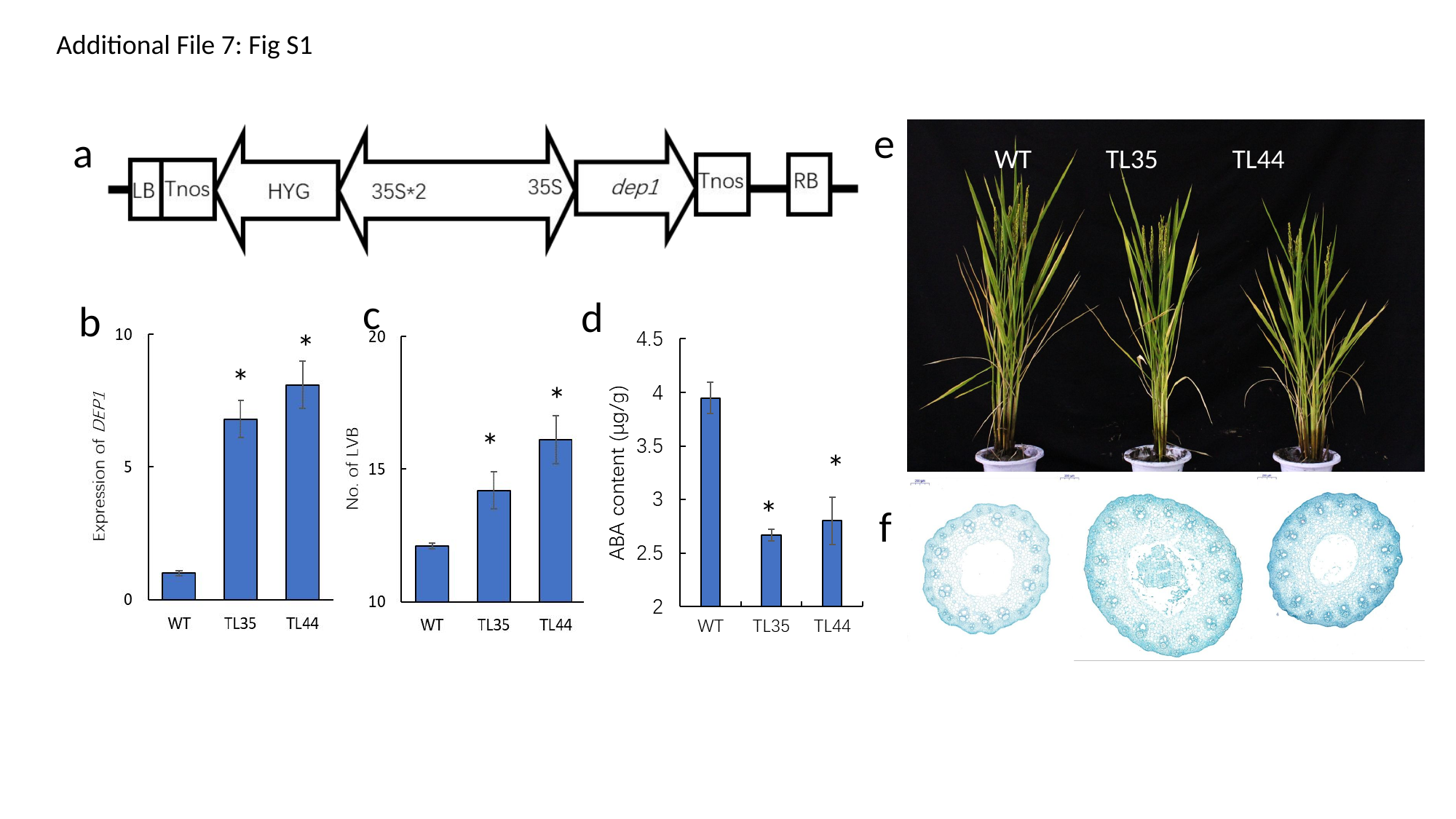

Additional File 7: Fig S1
e
a
WT TL35 TL44
c
d
b
*
*
*
*
*
*
*
f

Supplement: Supplementary file 5 — Figure S1. The overexpression of DEP1. (a) Overexpression construct for rice transformation. (b) The expression levels of TL35, TL44, and wild-type (WT). (c) The number of LVBs of TL35, TL44, and WT. (d) The endogenous ABA level of WT and dep1 overexpression lines. (e) The WT plant and the dep1 overexpression lines. (f) The difference in vascular bundles in WT plant and dep1 overexpression lines. (PPTX 53 kb) [file 12284_2019_278_MOESM5_ESM.pptx]

## Slide 1
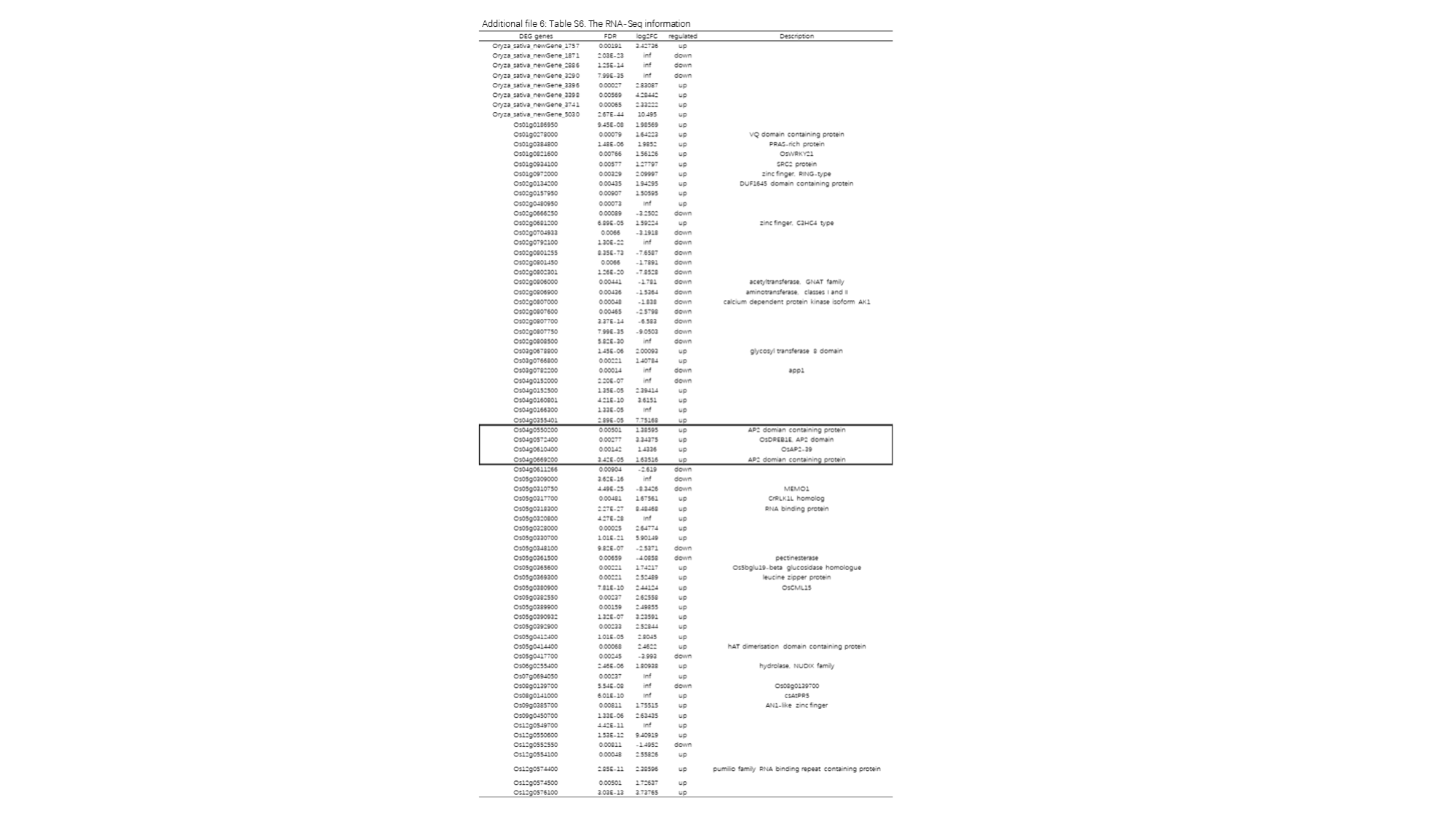

Supplement: Supplementary file 6 — Table S6. The RNA-seq information (PPTX 146 kb) [file 12284_2019_278_MOESM6_ESM.pptx]

## Slide 1
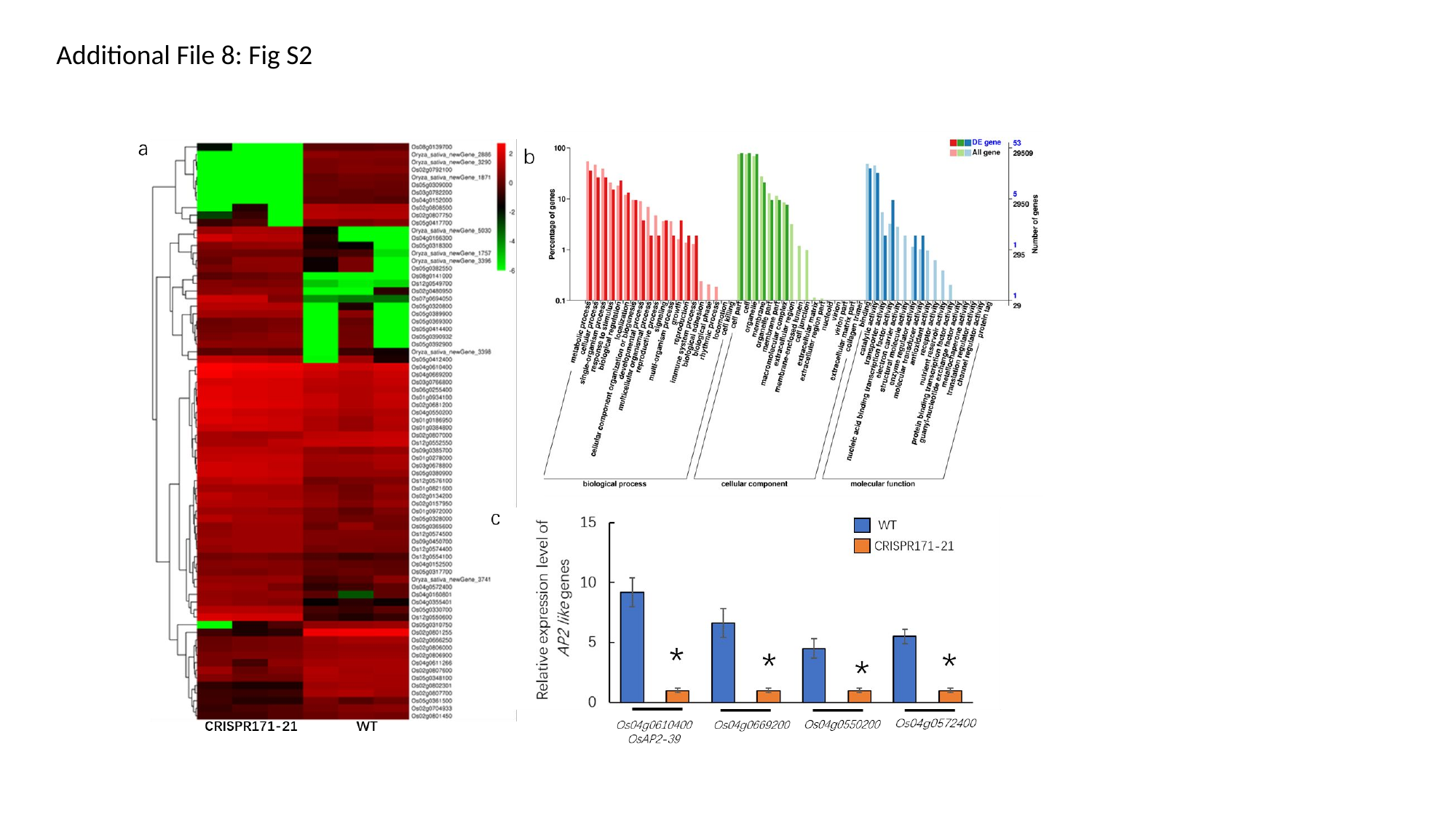

Additional File 8: Fig S2

Supplement: Supplementary file 7 — Figure S2. Expression patterns of DEGs. (a) Hierarchical clustering of DEGs. (b) GO analysis of the DEGs between CRISPR171–21 and wild-type plants. (c) The expression level of AP2-like genes in CRISPR171–21 and wild-type plants. (PPTX 3444 kb) [file 12284_2019_278_MOESM7_ESM.pptx]

## Slide 1
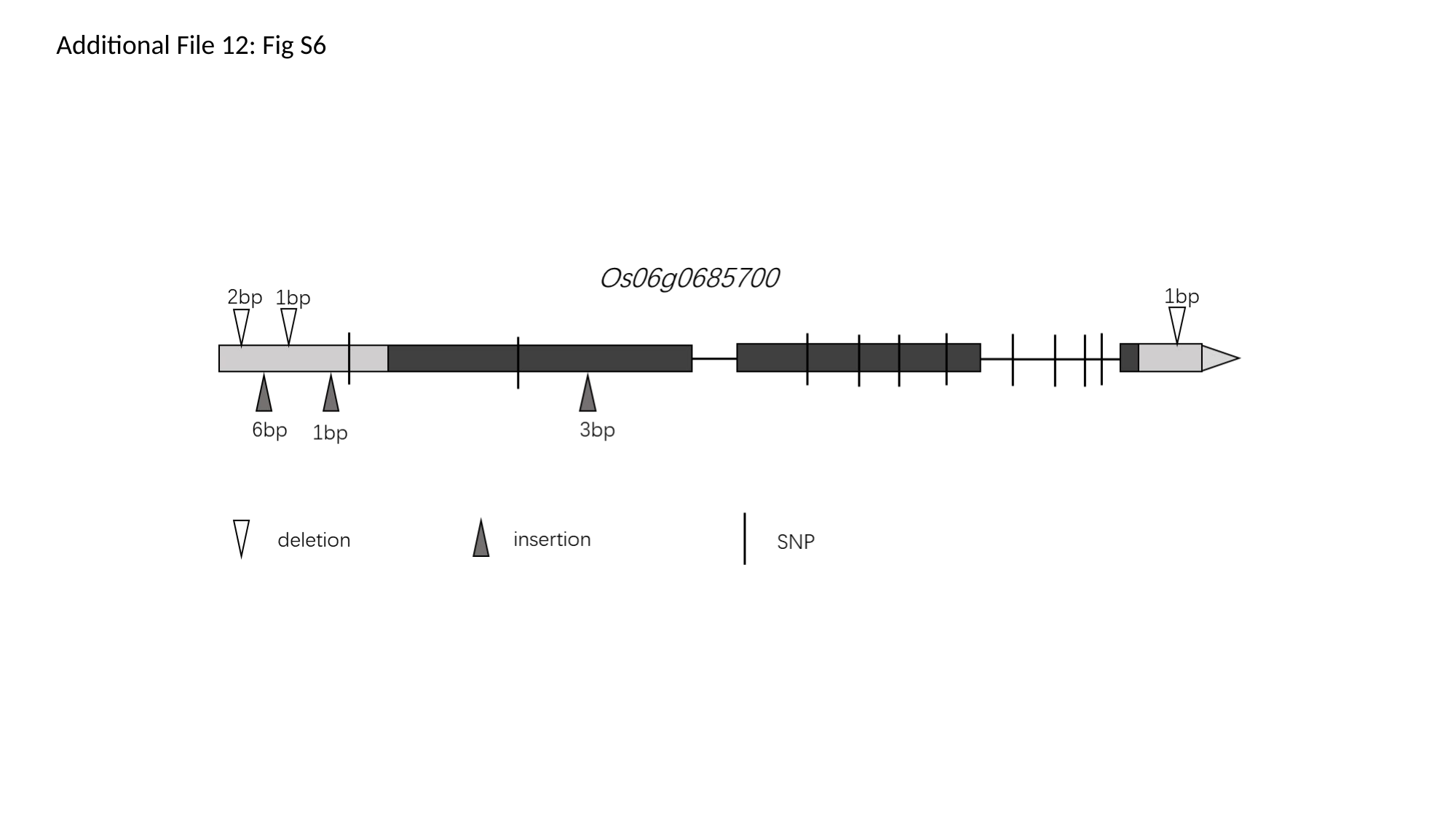

Additional File 12: Fig S6

Supplement: Supplementary file 11 — Figure S6. Os06g0685700 sequence differences between SN265 and R99 based on the sequence of SN265. (PPTX 46 kb) [file 12284_2019_278_MOESM11_ESM.pptx]

## Slide 1
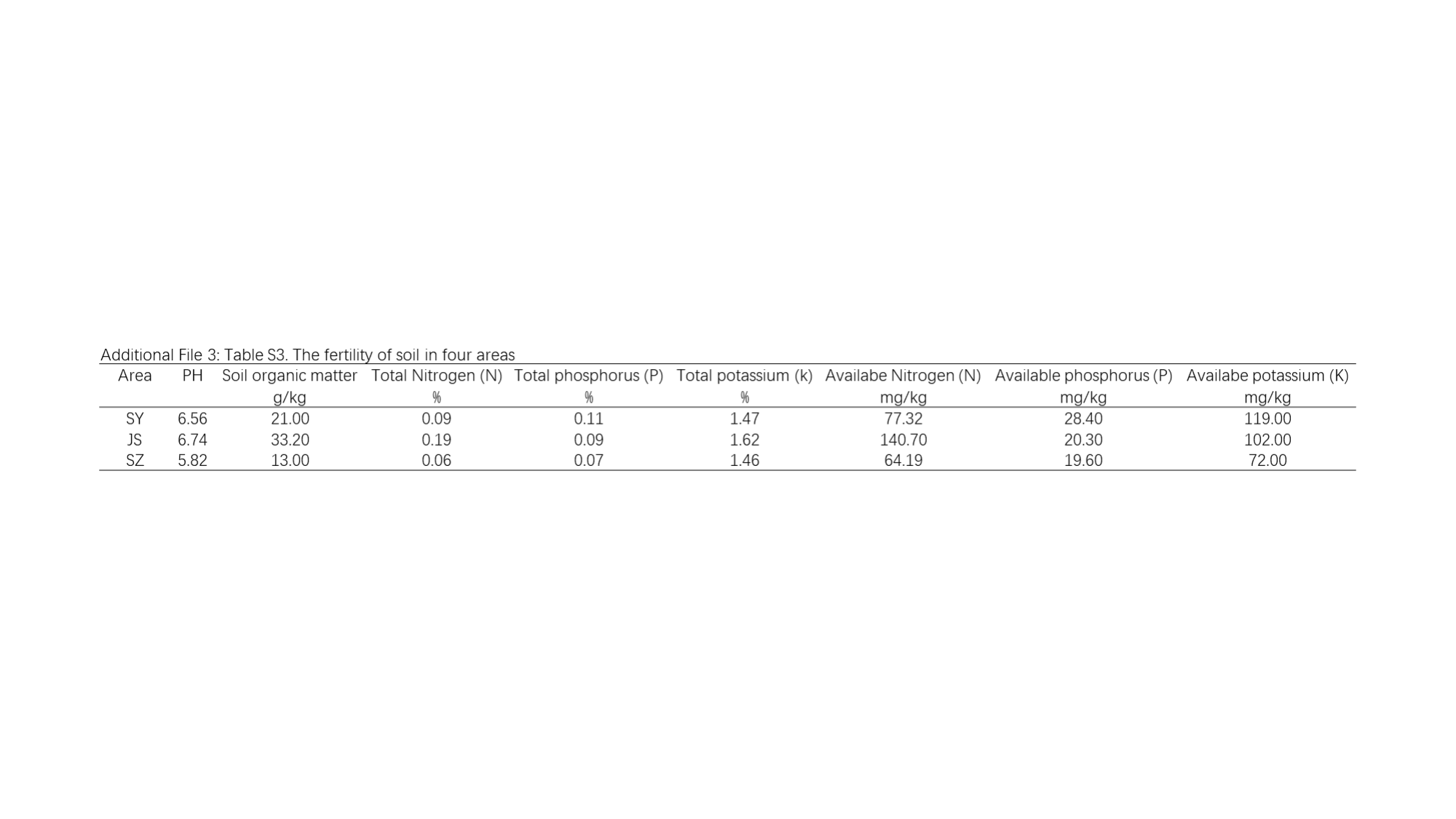

Supplement: Supplementary file 12 — Table S3. The fertility of the soil in three areas (PPTX 57 kb) [file 12284_2019_278_MOESM12_ESM.pptx]
