# Supplementary material for: Multiple areas investigation reveals the genes related to vascular bundles in rice
Source: Rice (N Y). 2019 Mar 21;12:17. doi: 10.1186/s12284-019-0278-x (PMC6428884; doi:10.1186/s12284-019-0278-x)
Supplement: Supplementary file 8 — Figure S3. DEP1 and OsAP2–39 expression levels in various organs of Sasanishiki. (PPTX 561 kb) [file 12284_2019_278_MOESM8_ESM.pptx]

## Slide 1
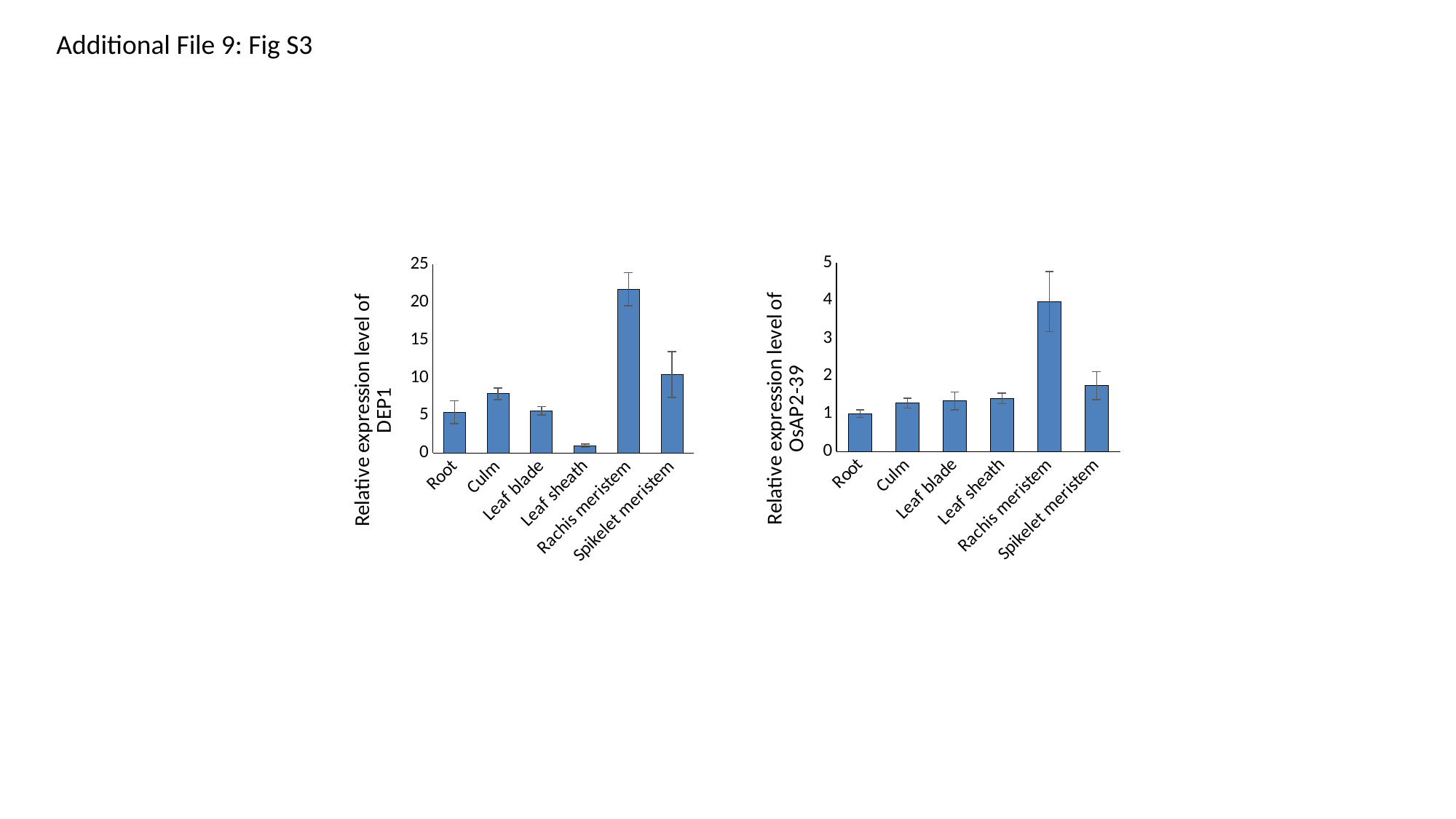

Additional File 9: Fig S3
### Chart
| Category | DEP1 |
|---|---|
| Root | 1.0 |
| Culm | 1.2836053145276607 |
| Leaf blade | 1.3430468535284534 |
| Leaf sheath | 1.4095889810761613 |
| Rachis meristem | 3.9730262607594238 |
| Spikelet meristem | 1.7455936652340682 |
### Chart
| Category | DEP1 |
|---|---|
| Root | 5.412948857453753 |
| Culm | 7.8576351109902065 |
| Leaf blade | 5.6077257889009795 |
| Leaf sheath | 1.0 |
| Rachis meristem | 21.703119332607905 |
| Spikelet meristem | 10.385201305767138 |
