# Supplementary material for: Multiple areas investigation reveals the genes related to vascular bundles in rice
Source: Rice (N Y). 2019 Mar 21;12:17. doi: 10.1186/s12284-019-0278-x (PMC6428884; doi:10.1186/s12284-019-0278-x)
Supplement: Supplementary file 9 — Figure S4. Germination investigation under exogenous ABA treatment. (PPTX 52 kb) [file 12284_2019_278_MOESM9_ESM.pptx]

## Slide 1
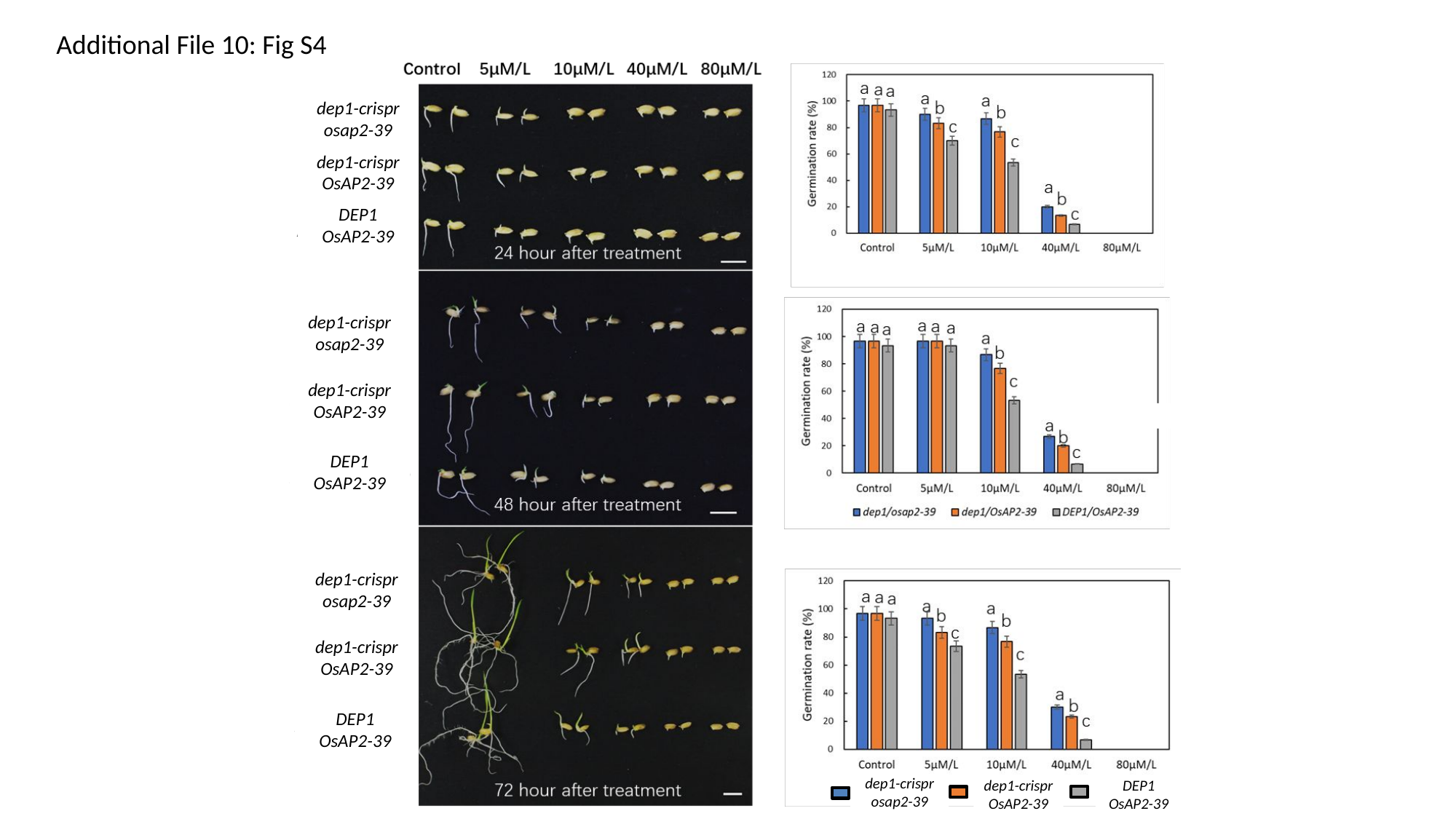

Additional File 10: Fig S4
dep1-crispr
osap2-39
dep1-crispr
OsAP2-39
DEP1
OsAP2-39
dep1-crispr
osap2-39
dep1-crispr
OsAP2-39
DEP1
OsAP2-39
dep1-crispr
osap2-39
dep1-crispr
OsAP2-39
DEP1
OsAP2-39
dep1-crispr
osap2-39
dep1-crispr
OsAP2-39
DEP1
OsAP2-39
