# Supplementary material for: Multiple areas investigation reveals the genes related to vascular bundles in rice
Source: Rice (N Y). 2019 Mar 21;12:17. doi: 10.1186/s12284-019-0278-x (PMC6428884; doi:10.1186/s12284-019-0278-x)
Supplement: Supplementary file 10 — Figure S5. The number of LVBs between control and ABA treatment plants, and the expression level of OsAP2–39 between control and ABA treatment plants. (PPTX 945 kb) [file 12284_2019_278_MOESM10_ESM.pptx]

## Slide 1
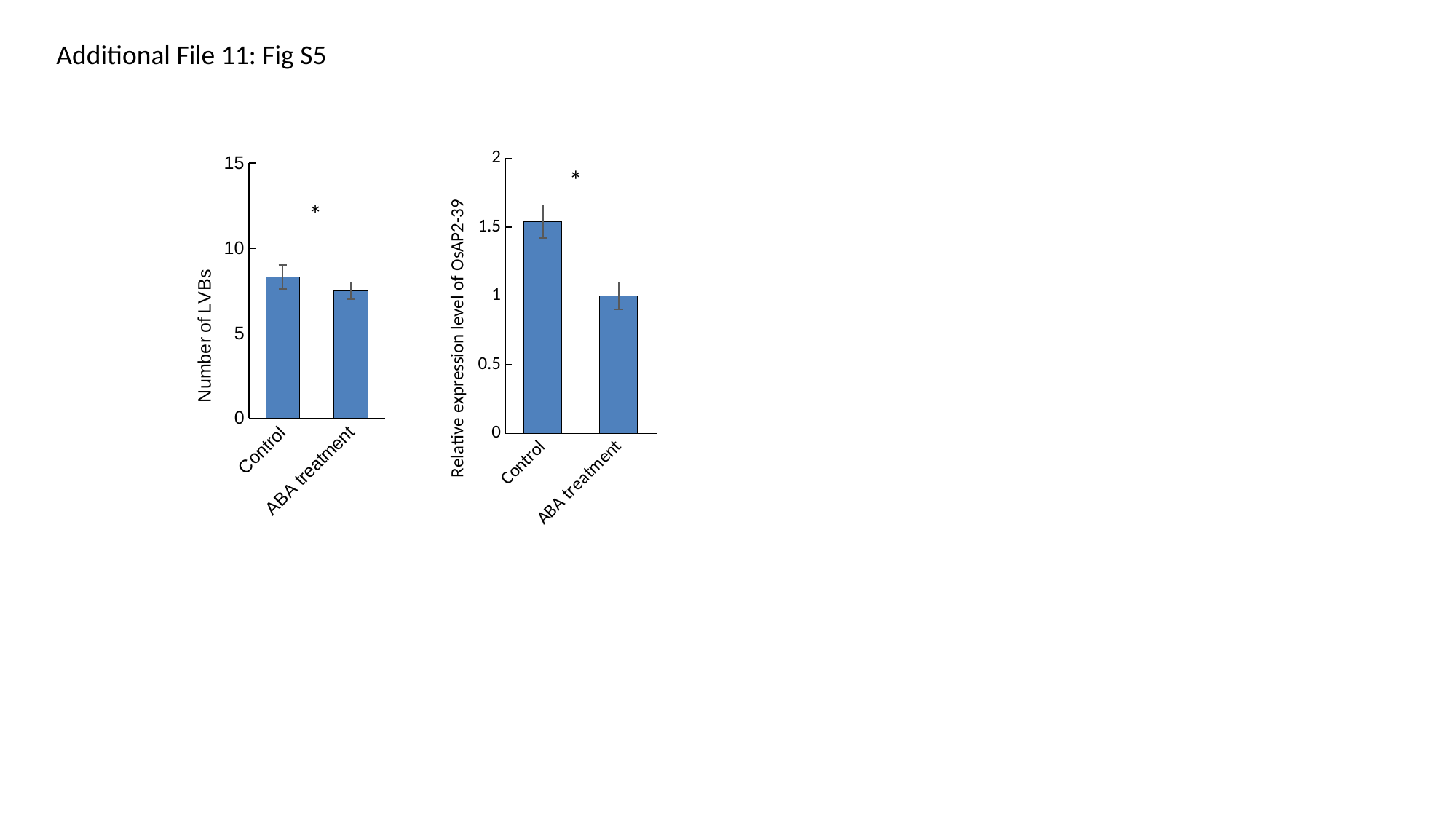

Additional File 11: Fig S5
### Chart
| Category | |
|---|---|
| Control | 1.54 |
| ABA treatment | 1.0 |
### Chart
| Category | |
|---|---|
| Control | 8.3 |
| ABA treatment | 7.5 |*
*
